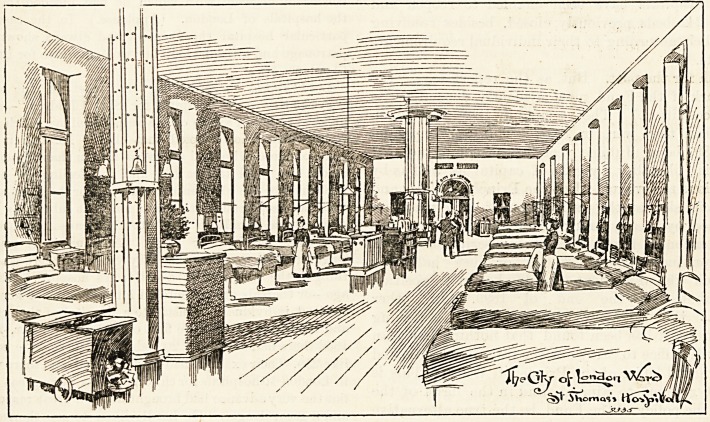# The Prince of Wales's Hospital Fund of London

**Published:** 1899-05-13

**Authors:** 


### THE PRINCE OF WALES'S HOSPITAL FUND OF LONDON.

No one can peruse the second annual report of the Council of the Prince of Wales's Hospital Fund dispassionately without acknowledging that it merits the warm support of all classes of the community. It has been weighed in the balance and has not been found wanting. The sagacious policy of the Council has silenced even carping critics, whilst the patience, the judgment, and the fairness of the Special Distribution Committee have confirmed the public in the belief that the money contributed to the Fund is in the hands of most capable administrators, and have convinced the managers of the hospitals that their true interests cannot fail to be promoted by steady adherence to the principles pursued.

In these circumstances the appeal which has been issued by the honorary secretaries will, we are sure, meet with a favourable response from many who have hitherto either underestimated the necessities of the hospitals or have not taken them into consideration. It is, however, more to those who have not so far subscribed to the Fund than to those who may be able to increase their subscriptions, that the appeal is addressed. The most important step initiated by the Council in 1898 was the appointment of a Visiting Committee, comprising prominent members of the medical profession, who are charged with the task of obtaining accurate information as to the condition and the needs of the hospitals. While it is open to all to apply for a grant, every application is inspected and reported upon by this committee of experts before an award is made by the Special Distribution Committee, who are thus placed in possession of the fullest knowledge available. The total sum distributed during the twelve months was £32,500, of which £23,200 was given as annual grants and the balance as donations. As an illustration of the care exercised in making the grants, it may be mentioned that most of them were conditional on the amount being used in each case for supplying some special needs, such as the opening of closed wards, the improvement of the accommodation for the nursing staff, the remedying of structural defects, or the relief of serious financial embarrassment. The details of the grants recommended by the Distribution Committee attest, in a striking manner, the conscientiousness with which they perform their arduous duties. To summarise the results achieved by the Fund in a sentence, it may be said that £89,000 has been distributed in such a way that the hospitals have been enabled to reopen and maintain 242 beds previously closed, besides receiving financial help according to their individual requirements and merits.

So much for the past. But, as the report says, "the future work of the Fund must evidently depend on its financial condition." The receipts in 1898 were much smaller than they were in 1897. This was expected, because 1897 being Jubilee year it was commemorated with special liberality by gifts of capital as well as by contributions to revenue. But the Prince of Wales and the Council ask that the enthusiasm of 1897 may now be replaced by steady and continuous support. The present income from annual subscriptions and interest produces about £25,000. or just half the sum which the President should be in a position to distribute at the end of 1899. In other words, as the outcome of two years' searching investigations, it has been found that nothing less than £50,000 will suffice to carry out the intentions indicated by the original letter of the Prince of Wales. This is not a vast revenue to aim at, and in the light of the fact that the policy of the Fund is the true alternative to the deplorable expedient of throwing the hospitals on the rates it is incredible that there will be insurmountable difficulties in obtaining it.

There is no question of rivalry with the Sunday and Saturday Hospital Funds. With these organisations the Prince and the General Council are working in cordial harmony. Their claim to support is indefeasible. It is to the hundreds of thousands, especially among the middle and working classes, who do not subscribe a farthing to the hospitals in any shape or form, that the Council especially look, as they have a right to look, for the means which alone can solve the urgent problem of the maintenance of the hospitals of London in a state of efficiency. The machinery for securing this great end is in active and successful operation; nothing is wanted but the exercise of a little self-denial, which would not be without its reward, on the part of that immense proportion of the public whose ears have hitherto been deaf to the cry of charities, the existence of which is the noblest product of our civilisation and the perpetual witness of our Christianity.

## Figures and Tables

**Figure f1:**